# Novel Insight into Neutrophil Immune Responses by Dry Mass Determination of *Candida albicans* Morphotypes

**DOI:** 10.1371/journal.pone.0077993

**Published:** 2013-10-30

**Authors:** Ava Hosseinzadeh, Constantin F. Urban

**Affiliations:** 1 Department of Clinical Microbiology, Umeå University, Umeå, Sweden; 2 Department of Molecular Biology, Umeå University, Umeå, Sweden; 3 Laboratory for Molecular Infection Medicine, Sweden (MIMS), Laboratory for Molecular Infection Medicine, Sweden; 4 Umeå Centre for Microbial Research, Umeå, Sweden; Leibniz Institute for Natural Products Research and Infection Biology- Hans Knoell Institute, Germany

## Abstract

The common fungal pathogen *Candida albicans* has the ability to grow as a yeast or as a hypha and can alternate between these morphotypes. The overall biomass of both morphotypes increases with growth. However, only yeasts, but not hyphae, exist as discrete cellular entities. Multiplicity of infection (MOI) is a useful parameter to determine the initial inoculum of yeasts for *in vitro* infection assays. Since the amount of hyphae is difficult to quantify, comparable starting conditions in such assays cannot be determined accurately for yeasts and hyphae using MOI. To circumvent this problem, we have established a set of correlation coefficients to convert fungal metabolic activity and optical density to dry mass. Using these correlations, we were able to accurately compare ROS production and IL-8 release by polymorphonuclear neutrophils upon infection with equal dry mass amounts of yeast and hyphal morphotypes. Neutrophil responses depended on the initial form of infection, irrespective of *C. albicans* wild-type yeasts transforming to hyphal growth during the assay. Infection with a high mass of live *C. albicans* yeasts resulted in lower neutrophil ROS and this decrease stems from efficient ROS detoxification by *C. albicans* without directly affecting the phagocyte ROS machinery. Moreover, we show that dead *C. albicans* induces significantly less ROS and IL-8 release than live fungi, but thimerosal-killed *C. albicans* were still able to detoxify neutrophil ROS. Thus, the dry mass approach presented in this study reveals neutrophil responses to different amounts and morphotypes of *C. albicans* and serves as a template for studies that aim to identify morphotype-specific responses in a variety of immune cells.

## Introduction

The incidence of fungal infection is heightened in immunocompromised individuals, who constitute the major risk group for severe mycoses [1]. Fungal pathogens are a common cause of death among this group [2]. *C. albicans* is the commonest human fungal pathogen and has the characteristic ability to interchange between two alternative growth morphologies [3], [4]. In the laboratory, exposing *C. albicans* to different culture conditions can induce morphological transition. In rich medium, at 30°C, the yeast form of growth is predominant, while in the presence of human serum, at elevated temperature or pH, filamentous forms germinate from yeast mother cells and pseudohyphae or true hyphae are generated [5]. Several *in vitro* and *in vivo* models have demonstrated that the hyphal form is required to invade tissue and disseminate more deeply within the body [6], [7]. *C. albicans* mutant strains that cannot form hyphae are often severely attenuated in virulence, such as the *Δefg1* mutant, which is deficient in the transcription factor *EFG1* required for filament formation [8]. On the other hand, a *Δtup1* mutant strain, deficient in the transcriptional repressor of filamentous growth (*TUP1*), grows constitutively as pseudohyphal and hyphal forms [9]. Interestingly, although the hyphal forms are essential for invasion, the *Δtup1* mutant is avirulent in infection models [9]. In biopsies from human patients, multiple growth forms are usually detected [10]. Thus, reversibility of morphotype transition might be an important pathogenic adaptation [7]. To understand the contribution of individual growth morphologies to the mechanisms of *C. albicans* pathogenicity it is essential to compare host responses to different morphological growth forms. It has been shown that host cells respond differentially to yeast-form and to hyphal cells. For instance, dendritic cells produced different amounts of interleukin-12 (IL-12) and IL-14 upon recognition of different *C. albicans* morphotypes [11]. Also, polymorphonuclear leucocytes (neutrophils) migrate more rapidly towards hyphal cells and neutrophil extracellular signal-regulated kinase (ERK) signalling results in specific killing of *C. albicans* filaments [12]. In oral epithelial cells, a biphasic innate immune mitogen-activated protein kinase (MAPK) response results in specific recognition of *C. albicans* hyphae [13], [14]. Earlier studies investigating the sensing of *C. albicans* pathogen-associated molecular patterns in monocytes and macrophages, focused either on live or dead *C. albicans*, and employed one or a few different MOIs and time points [12], [15]. Dead microbes are often used to investigate microbe-host cell interaction to prevent side effects due to microbial overgrowth. Considering that *C. albicans* yeasts change morphology to hyphae under cell culture conditions used in immune cell assays, it is particularly appealing to be able to use dead microbes in the respective growth morphology. Therefore, we chose to study how neutrophils, the first line of the cellular defence against microbes, respond to *C. albicans* with the aim of discriminating between responses towards different growth forms and towards viable and non-viable *C. albicans*. Upon bacterial or fungal recognition, neutrophils utilize arrays of defence mechanisms to clear the infection. Two of the key functions are the production of reactive oxygen species (ROS) and IL-8 secretion. Released IL-8 recruits new neutrophils to the site of infection and mediates neutrophil activation [16]. ROS serve to mediate inflammatory signalling and to destroy pathogens [17]. To induce the oxidative burst that generates ROS, external signals converge to activate protein kinase C (PKC) that eventually leads to the assembly and activation of the nicotinamide adenine dinucleotide phosphate (NADPH) oxidase complex. Molecular oxygen is reduced to superoxide, which reacts further to form other antimicrobial ROS such as H_2_O_2_ or HOCl. To prevent damage by ROS, *C. albicans* has evolved several detoxification mechanisms, mainly utilizing superoxide dismutases (SOD) and catalases [18], [19], [20]. SOD*s* dismutate superoxide anions into molecular oxygen and hydrogen peroxide and paradoxically high SOD activity prevents the formation of higher amounts of hydrogen peroxide by other reactions [21]. There are six genes encoding SODs in *C. albicans*; two cytoplasmic (*SOD1*, *SOD3)*, one mitochondrial (*SOD2),* and three cell surface-localized (*SOD4*, *SOD5* and *SOD6)* [19]. Extracellular SODs detoxify neutrophil-derived [20], as well as macrophage- and dendritic cell-derived, ROS [18]. Among extracellular SODs, *SOD5* expression is up-regulated following contact with neutrophils [22].

To study immune cell responses, it is widely accepted that multiplicity of infection (MOI) is a convenient parameter with which to determine a ratio of microbes to host cells. However, MOIs of pleomorphic microorganisms, such as *C. albicans*, are difficult to assess and to compare. While microscopic counting of yeast cells and short hyphae is feasible, it is more challenging to assess accurate numbers of larger hyphae that tend to clump. Additionally, hyphae increase in size considerably during apical growth although total cell number remains unchanged. In contrast, yeast cells proliferate by cytokinesis and increase in number exponentially. Therefore, comparative MOI-based approaches do not ensure the application of equal biomasses of yeast and hyphal forms. To be able to study neutrophil-*Candida* interaction in more detail, we applied correlation tools to directly compare different growth forms or different strains of *C. albicans* by using dry mass (DM) as a common denominator for different *C. albicans* morphotypes. The correlation factors enabled us to convert between different measurements, such as cell metabolic activity (MA), optical density (OD) and DM. We applied these correlations to study differential neutrophil responses and demonstrated that human neutrophils elicit a wide-range pattern of ROS production and IL-8 secretion upon stimulation with *C. albicans*. Whether or not the overall neutrophil response is higher towards hyphae than towards yeast cells depends strictly on the amount of biomass used for the initial infection. Due to the requirement for different growth conditions to induce either yeasts or hyphae it remains unknown what contribution morphology-independent factors might have on the neutrophil responses observed here. Notwithstanding this, our approach describes the complex relationship of morphotype and total amount of *C. albicans* biomass in tuning neutrophil responses with unprecedented precision. The study presented here should serve as the basis for further investigations of this kind possibly with other pleomorphic microorganisms.

## Materials and Methods

### Ethical Statement

Blood of healthy volunteers was obtained according to the recommendations of the local ethical committee (Regionala etikprövningsnämnden i Umeå) as approved in permit Dnr 09–210 M with fully informed written consent of the donors. All investigations were conducted according to the principles expressed in the Declaration of Helsinki.

### *C. albicans* Strains, Media and Microbial Culture

The wild-type *C. albicans* clinical isolate SC5314 [23], *Δefg1* mutant [24], *Δtup1* mutant, *Δsod5* mutant, *Δsod5::SOD5* revertant, *Δsod4Δsod5Δsod6* triple mutant [18], and *Saccharomyces cerevisiae* wild-type strain UMY2067 were cultured overnight in 20 ml YPD (1% yeast extract, 2% bacto peptone, 2% glucose) at 30°C on an orbital shaker at 300 rpm. To grow *C. albicans* in the yeast morphotype, 20 ml YPD fresh growth medium was used to dilute the overnight culture and incubation was continued at 30°C. To induce hyphae, the cells were inoculated in 20 ml RPMI medium (RPMI 1640, Lonza) and incubated at 37°C. The starting OD_600 nm_ for all subcultures was 0.1, except for the *Δtup1* mutant. This constitutively filamentous strain was difficult to handle. To inoculate cultures we used wet mass (pellet after centrifugation, 50 mg in 20 ml YPD) to obtain approximately similar starting amounts. For inactivation, *C. albicans* was incubated for 1 h at 65°C (heat-killed) or overnight in the presence of 0.01% thimerosal (Sigma-Aldrich) at 4°C (thimerosal-killed). Killed *C. albicans* cells were washed three times with phosphate-buffered saline (PBS) to remove excess thimerosal. The viability of the culture was determined by plating on YPD agar medium.

### Isolation of Human Neutrophils

Neutrophils were isolated by density gradient centrifugation from healthy volunteers as previously described [25]. Briefly, neutrophils were isolated by layering blood on top of Histopaque (1119 from Sigma Aldrich) followed by a continuous Percoll (GE Healthcare) gradient. The isolated cells were stained with trypan blue and viable cells were counted in a Neubauer chamber.

### OD Measurement

Yeast growth was measured spectrophotometrically (DU 730 UV/Vis, Beckman Coulter). At indicated time points, the liquid yeast culture was diluted and OD was measured at 600 nm in a plate reader (FLUOstar Omega, BMG Labtech). The cell number of *S. cerevisiae* and *C. albicans* strains was determined using the correlation factor 3×10^7^ cells/ml at OD_600_ = 1.

### Cell Viability Measurement by XTT

Mitochondrial dehydrogenase in metabolically active cells reduces the tetrazolium dye 2,3-bis-(2-methoxy-4-nitro-5-sulfophenyl)-2H-tetrazolium-5-carboxanilide (XTT, Invitrogen) to a water-soluble formazan product [26]. The amount of product reflects the amount of viable fungal cells independently from their growth form. At the indicated time points, liquid *C. albicans* cultures were washed twice with PBS and 200 µl of *C. albicans* suspensions were added into 24-well tissue culture plates (BD Falcon). The same volume of XTT solution (1 mg/ml XTT and 80 µg/ml Coenzyme Q in PBS) was added. The plates were incubated at 37°C for 20 min. After incubation, the plates were spun for 5 min at 1500×g and the supernatants were transferred into clear sterile 96-well plates (BD Falcon). The OD at 450 nm was determined from serial dilutions in PBS (FLUOstar Omega, BMG Labtech). The liquid culture flasks were kept on ice for further OD or DM measurements.

### *C. albicans* DM Measurement

To determine the DM, 20 ml *C. albicans* liquid culture were loaded on top of 2.5 cm pre-weighted, circular glass microfiber filters (Whatman, 1.2 µm pore size). The fluid was removed by vacuum filtration. The filters were rinsed several times with PBS and dried in capped glass petri dishes in an oven at 80°C for 24 h. The exact liquid culture volume loaded on each filter was recorded to obtain the DM per volume.

To calculate the DM of single cells, DM was plotted versus time and the yeast cell number was additionally determined by counting in a Neubauer chamber at each time point (data not shown). The number of cells was then calculated for a DM of 1 µg. The calculations were performed for hyphae with the assumption that all *C. albicans* yeast cells germinate under hyphae-inducing conditions.

### *C. albicans* Cell Surface Area and Volume Measurements

Immunostainings were prepared from *C. albicans* yeast and hyphal liquid cultures using antibodies directed against *C. albicans* cell wall components (Rabbit anti-*Candida*, polyclonal antibody, Acris, 8 µg/ml). We used a confocal microscope (Nikon C1 confocal microscope) and image software (NIS-Elements AR, version 3.2.0) to determine *C. albicans* cell dimensions (length, width and depth) at different time points (Fig. S1). To calculate the yeast and hyphal mother cell surface area we applied the Knud Thomsen surface area formula for ellipsoid objects: S = 4π [(a^p^b^p^+a^p^c^p^+b^p^c^p^)/3]^1/p^ (p = 1.6075, a = radius of length, b = radius of width axis, c = radius of depth). To determine the germ tube cell surface area we applied the cylinder surface area formula: S = 2(πr^2^) +2(πrh) (r = radius of the length and h = height of the cylinder). This resulted in calculated cell surface areas of single yeasts or hyphae at different time points during culturing. Cell volumes were measured by applying formulas for ellipsoid objects (V = 4/3πabc) and cylindrical objects (V = πr^2^h) (a = radius of length, b = radius of width axis, c = radius of depth and h = height). For each single value at least 50 measurements were performed and the cell surface area and volume was calculated accordingly. Notably, to determine the cell surface area or volume of hyphae the calculated values for mother cells and germ tubes were summed.

### Measurement of ROS Production by Neutrophils

Human neutrophils were seeded into white 96-well plates (Nunc) in RPMI and incubated with 50 µM luminol (Sigma-Aldrich) and 1.2 u/ml horseradish peroxidise (Sigma-Aldrich) for 15 min at 37 °C and 5% CO_2_. Upon infection, luminescence generated by the production of ROS was measured every 2 min in triplicate for a total of 3 h in a luminometer (Infinite 200, TECAN). ROS quantification is either presented as relative light units (RLU) over time or as total ROS by calculating the area under the curve (AUC) in the given time period. For both types of measurements, background values from unstimulated cells were subtracted. Before use in ROS assays, *C. albicans* strains were sub-cultured for 3 h either at 30°C in YPD or at 37°C in RPMI and washed twice in PBS.

### ROS Scavenging Assay

Hydrogen peroxide (0.5 mM), 50 µM luminol and 1.2 u/ml horseradish peroxidise (all from Sigma-Aldrich) dissolved in RPMI, were added to white 96-well plates (Nunc) and incubated for 5 min at 37 °C and 5% CO_2_. Subsequently, different masses, morphotypes and strains of *C. albicans*, medium alone, or neutrophils were placed in individual wells. RLU was measured every 2 min in triplicate for a total of 20 min in a luminometer (Infinite 200, TECAN). Before use in ROS scavenging assays, *C. albicans* strains were sub-cultured for 3 h either at 30°C in YPD or at 37°C in RPMI and washed twice in PBS.

### IL-8 Release of Neutrophils

Human neutrophils were infected with *C. albicans* in triplicate. After 6 h incubation supernatants were collected and stored at -80°C. The IL-8 concentrations per 10^5^ neutrophils were determined using standard human IL-8 ELISA kit (ELISA MAX Deluxe, Biolegend) in a plate reader (FLUOstar Omega, BMG Labtech) in triplicate. Before use in IL-8 assays, *C. albicans* strains were sub-cultured for 3 h either at 30°C in YPD or at 37°C in RPMI and washed twice in PBS.

### Statistical Analysis

GraphPad Prism version 5.00 was used to plot all graphs, to analyze the correlations by regression analysis and to determine the respective R-squared values. One-way ANOVA followed by Bonferroni post-hoc comparison tests were performed for statistical analyses in Figure 4A and Figure S4A using the same software.

## Results

### Usage of *C. albicans* DM Enables more Accurate Determination of Neutrophil Responses in Dependence on Amount and Morphotype of *C. albicans*

During filamentous growth cell mass, surface and MA increase, however, the number of discriminable cellular units does not. An MOI approach used to assess the response of immune cells upon infection with filamentous and yeast forms of *C. albicans* might, therefore, be hampered. The cell surfaces of host and microbial cells are the contact sites during host-pathogen interactions and thus cell surface areas determine the quantity of these interactions. We hypothesized that DM, as opposed to MOI, is directly correlated to the cell surface area of the microbial entity. To confirm this, we determined the DM per *C. albicans* yeast and hypha at different time points after inoculation (Table 1). The mass of one yeast cell remained relatively constant, albeit showing a reduction from 35 pg for stationary-, down to 21 pg for exponentially-growing yeast cells (Table 1). In contrast, mass per hypha steadily increased after inoculation. Furthermore, we determined the cell surface area and volume of yeast and hyphal cells (Table 1) by measuring cellular dimensions at different growth time points microscopically (Fig. S1). Cell surface areas are presented as a ratio of cell surface area per µg *C. albicans* yeast versus hypha. Interestingly, this ratio remained constant over time ranging between 0.9 and 1.3. In contrast, the cell number ratio for yeast vs. hyphae increased from 1 to 3.6 during the same period, demonstrating that surface area directly correlates to DM. Our findings are in accordance to previous data [18] describing that 1 µg DM of *C. albicans* yeasts corresponds to 4×10^4^ cells (Table 1). Therefore, the DM approach permits infection of neutrophils with equal microbial surface areas. We next measured the DM of wild-type *C. albicans* grown as yeasts or hyphae, a yeast-locked *Δefg1* mutant and the constitutively filamentous *Δtup1* mutant (Fig. S2). According to our expectations, we observed exponential increase of the DM within 5 h of incubation for the *C. albicans* yeast forms (Fig. S2A) and the *Δefg1* mutant (Fig. S2C). Similarly, during hyphal growth, wild-type *C. albicans* (Fig. S2B) and the *Δtup1* mutant (Fig. S2D) gained mass in an exponential manner, albeit at slower rates. Resulting measurements were in accordance to previous attempts to quantify *C. albicans* DM (Table 1) [18], [27], [28].

**Table 1:** DM, cell surface area per *C. albicans* yeast or hypha and their ratios.

time (min)	mass of oneyeast (pg)	mass of onehypha (pg)	volume of oneyeast (µm^3^)	volume of onehypha (µm^3^)	surface area of oneyeast (µm^2^)	surface area of onehypha (µm^2^)
60	34.7±1.9	35.2±0.5	143.2±10.3	136.6±10.8	142.4±6.6	137.2±7.6
120	29.2±1.6	44.9±0.6	125.8±3.0	147.5±8.9	132.5±2.2	158.3±13.3
180	24.6±1.4	57.3±0.8	92.2±1.9	177.4±4.6	108.4±1.5	300.6±5.7
240	20.6±1.1	73.0±1.0	89.9±2.2	180.1±10.7	106.2±1.7	319.9±16.8
**time (min)**	**surface area** **(µm^2^)/1 µg yeast**	**surface area** **(µm^2^)/1 µg hypha**	**surface area ratio in** **1 µg (yeast: hypha)**	**yeast number/1 µg**	**hypha number/1 µg**	**cell number ratio in** **1 µg (yeast: hypha)**
60	4.2×106	3.9×106	1.1	2.9×104	2.8×104	1.0
120	4.6×106	3.5×106	1.3	3.5×104	2.2×104	1.6
180	4.5×106	5.2×106	0.9	4.1×104	1.7×104	2.4
240	5.2×106	4.4×106	1.2	4.9×104	1.4×104	3.6

Table 1: To determine the DM per yeast cell at the indicated time points the correlation coefficient factors of DM versus time and cell number versus time were used. With these values the average yeast concentration (DM per ml) was divided by the total number of yeast cells per ml. Similarly, calculations were performed for hyphae. Cell numbers of the initial inoculum at OD 0.1 were used assuming that under hypha-inducing conditions each yeast cell germinates and grows as hypha. To calculate the cell surface area and volume of yeasts and hyphae at different time points, the dimensions (length, width and depth) of approximately 50 immune-stained cells were measured by confocal microscopy as described in materials and methods. The ratio of cell surface areas per µg DM and cell number per µg DM from yeasts and hyphae were calculated for different time points. Data are presented as average of three biological replicates ± SD.

However, as the complete procedure to determine DM takes approximately two days, microbial amounts cannot be determined for subsequent infection experiments in this fashion. Therefore, we correlated DM to MA and OD to create a set of formulas that enabled us to interchange between these denominators (Table 2 and Fig. S3). MA was measured by reduction of XTT [26], [29]. OD was assessed spectrophotometrically. For each strain used, we calculated correlation equations for the parameter pairs DM/OD, DM/MA and MA/OD (Table 2). The correlations were highly reproducible and the linear regression resulted in R^2^ values between 0.87 and 0.97. Altogether, our correlations are suitable to describe a growing *C. albicans* culture and allow accurate adjustment of equal starting masses for comparative experiments.

**Table 2:** *C. albicans* morphotype and strain-specific correlation factors.

correlation type	slope (m)	Y-Intercept (b)	R-square	correlation range
**wild-type ** ***Candida albicans*** **, YPD, 30°C**				
**DM/OD**	0.8059±0.0485	0.03971±0.0140	0.9616	OD: 0.1–0.6
**DM/MA**	0.03611±0.0041	0.02641±0.0273	0.8767	MA: 3–16
**MA/OD**	20.74±1.4820	0.7621±0.4266	0.9468	OD: 0.1–0.6
***Δ efg1 Candida albicans*** **, YPD, 30°C**				
**DM/OD**	0.6756±0.0320	0.08627±0.0157	0.9717	OD: 0.1–0.9
**DM/MA**	0.08397±0.0058	0.02412±0.0266	0.9408	MA: 1.5–10
**MA/OD**	7.803±0.3698	0.8342±0.1819	0.9716	OD: 0.1–0.9
**wild-type ** ***Candida albicans*** **, RPMI, 37°C**				
**DM/MA**	0.01134±0.0010	0.03874±0.0122	0.9167	MA: 5–21.5
***Δ tup1 Candida albicans*** **, YPD, 30°C**				
**DM/MA**	0.04755±0.0048	0.1269±0.0452	0.8924	MA: 3–16.5

Table 2: Basic linear equations (y = mx±b) are presented to calculate DM. For yeast all three types of correlations: DM/MA, DM/OD and MA/OD are used. For the hyphal growth form the DM/MA correlation factor is proposed. The valid range of OD and MA measurements per 1 ml are indicated. The equations and R-square (R^2^) values were obtained from plotted graphs.

**Figure 1:**
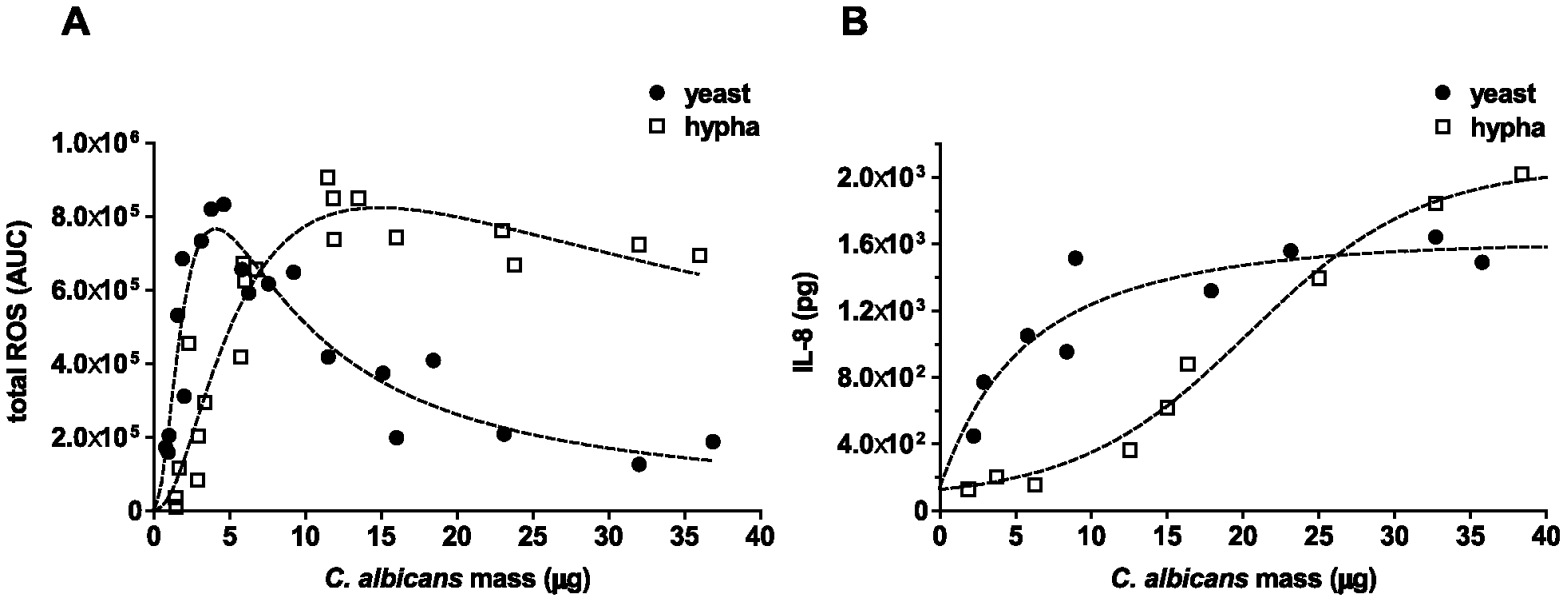
Neutrophil ROS and IL-8 response triggered with different amounts and morphotypes of wild-type *C. albicans*. Neutrophil ROS generation was measured in a luminol-based assay. Different DM amounts of *C. albicans* were used to infect 10^5^ neutrophils. Each data point represents AUC corresponding to the total ROS in the course of 3 h (A). IL-8 secretion was measured from supernatants of 10^5^ neutrophils after 6 h by ELISA (B). Neutrophils were infected with *C. albicans* yeasts (closed circles) or hyphae (open squares). The dashed line represents the best-fit curve of three individual assays from different donors. Masses to MOI conversion for 10^5^ neutrophils: (Yeasts) 1 µg≙0.4, 5 µg ≙2.1, 10 µg≙4.1, 20 µg≙8.2, 30 µg≙12.3; (hyphae) 1 µg≙0.2, 5 µg≙0.9, 10 µg≙1.7, 20 µg≙3.4, 30 µg≙5.1.

We applied the mass correlations in infection experiments with human neutrophils using different *C. albicans* strains and growth forms. Neutrophil ROS were measured in a luminol-based assay over 3 h and thus, each data point represents the complete ROS production over a 3 h time period from one individual experiment (Fig. 1A). As expected, total ROS production of neutrophils heightened with increasing infection doses of *C. albicans* yeasts (Fig. 1A). However, ROS production reached a maximum at a DM infection dose of 4–5 µg and declined rapidly with further increases in the mass of yeasts. This indicates that *C. albicans* yeasts can either suppress ROS production in human neutrophils, as it has been suggested earlier for different mouse and human phagocytes [30], or have the ability to detoxify ROS by means of antioxidant enzymes, as described previously [18]. Notably, when we added pre-grown *C. albicans* hyphae, the ROS response pattern was very different from the pattern induced by yeast cells. Larger amounts were required to induce significant amounts of ROS (Fig. 1A). Beyond 2–3 µg of hyphae, neutrophil ROS production was increased. At approximately 7 µg, the amount of ROS induced was similar for both yeasts and hyphae. In contrast to yeast-induced ROS, hyphae-induced levels did not decline with increasing infection dose, but rather rose towards a maximum. Only at very high DM of hyphae (>25 µg/10^5^ neutrophils) did neutrophil ROS decline slightly below maximum. Taken together, neutrophil ROS production depends largely on the initial infection dose of *C. albicans* as well as on the growth form. Thus, at low *C. albicans* to neutrophil ratios, yeasts induce more ROS than hyphae and at high *C. albicans* to neutrophil ratios, stimulation with hyphae resulted in more ROS than induction by yeasts.

IL-8 secretion is an important response of human neutrophils upon various stimuli [31]. *C. albicans* infection triggers IL-8 release by neutrophils [12]. We infected human neutrophils with different doses, morphotypes and strains of *C. albicans* similarly as described above and quantified IL-8 in the supernatants 6 h post infection (p.i.). Notably, we observed similarities in the pattern for IL-8 secretion and ROS production (Fig. 1A and B). At infection doses below 20 µg, *C. albicans* yeasts induced more IL-8 secretion than hyphae up to a maximum value of approximately 1.5 ng/ml (Fig. 1B). However, at masses above 25 µg, hyphae induced up to 40% more IL-8 secretion than yeasts (Fig. 1B). In contrast to ROS production, IL-8 release by neutrophils did not decline by stimulation with high masses of *C. albicans* yeasts indicating that *C. albicans* might not degrade IL-8. We conclude that the infection dose defines whether *C. albicans* hyphae or yeasts trigger more neutrophil IL-8 secretion.

### *C. albicans* Strains Arrested in Yeast or Hyphal Growth Induce Similar Neutrophil ROS Responses as the Respective Morphotypes of Wild-type *C. albicans*

**Figure 2:**
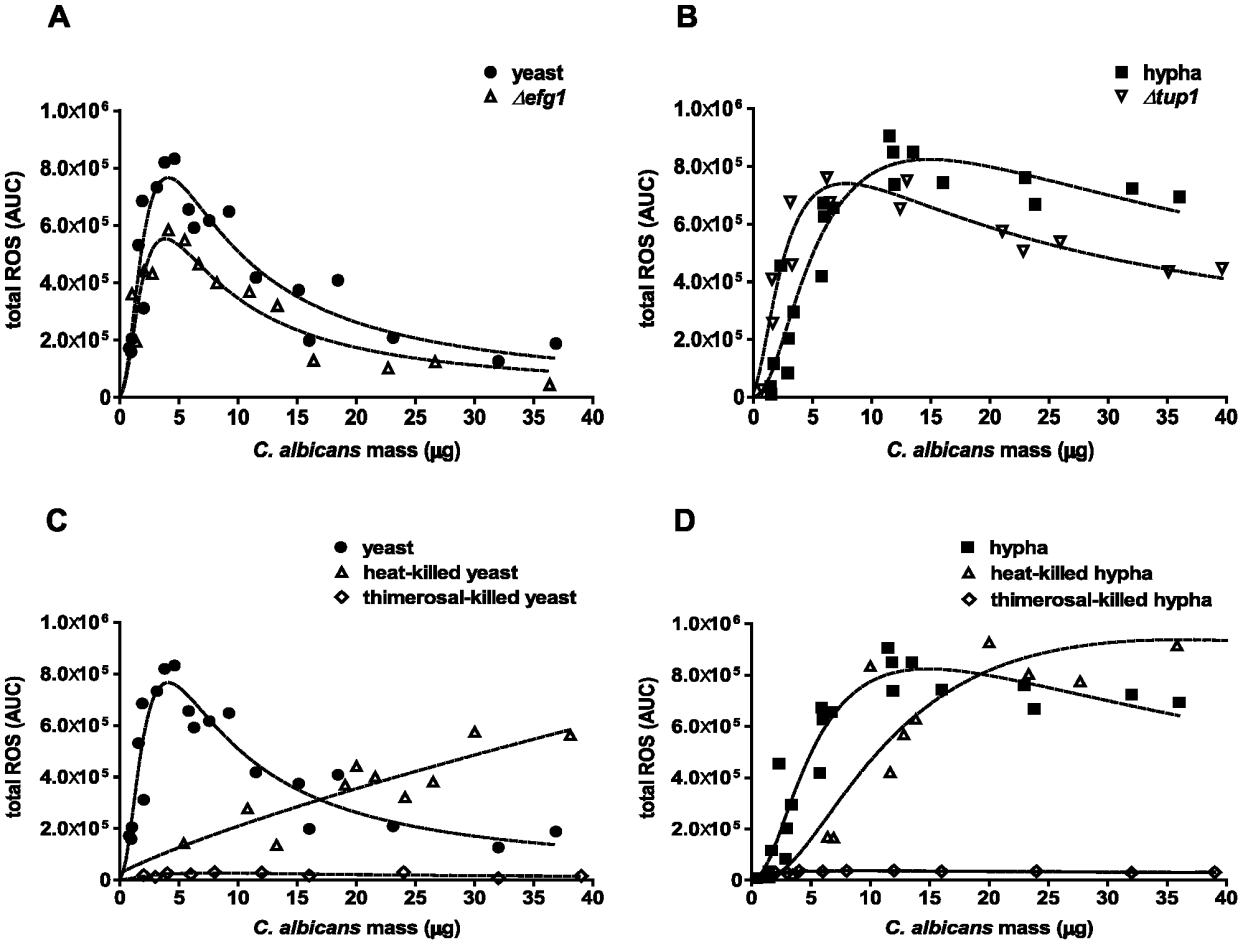
Neutrophil ROS response pattern depends on amount, morphotype and viability of *C. albicans*. Neutrophil ROS generation was measured in a luminol-based assay. Different DM amounts of *C. albicans* were used to infect 10^5^ neutrophils. Each data point represents AUC corresponding to the total ROS in the course of 3 h. Neutrophils were infected either with wild-type yeasts (closed circles) or *Δefg1* mutant (open triangles) (A) or wild-type hyphae (closed squares) or *Δtup1* mutant (open inverted triangles) (B). Neutrophils were infected with heat-killed (open triangles) or thimerosal-killed (open rhombuses) yeasts (C) or hyphae (D). The dashed line represents the best-fit curve of three individual assays from different donors. Masses to MOI conversion for 10^5^ neutrophils: (Yeasts) 1 µg≙0.4, 5 µg≙2.1, 10 µg≙4.1, 20 µg≙8.2, 30 µg≙12.3; (hyphae) 1 µg≙0.2, 5 µg≙0.9, 10 µg≙1.7, 20 µg≙3.4, 30 µg≙5.1.

We next aimed to elucidate the different neutrophil ROS response patterns upon infection with yeast and hyphal morphotypes in more detail. Of note, under the conditions of the immune assay, *C. albicans* yeasts form germ tubes. Thus, a neutrophil response could be largely influenced by newly forming hyphae. To address this, we investigated the contributions of the two morphotypes to shape the neutrophil response by infection with morphotype-locked mutant strains of *C. albicans*. We used the *Δefg1* mutant strain that is defective to form hyphae under these conditions [9]. The total ROS production of neutrophils upon different infection doses was very similar in response to the *Δefg1* mutant strain compared to wild-type yeasts (Fig. 2A). We observed a sharp rise of ROS at low masses, a maximum at approximately 5 µg and a decline in measurable ROS above 10 µg *Δefg1* infection dose. However, the maximum resulting ROS signal was overall slightly lower than with wild-type yeasts, which is in good agreement to a previous report [32]. Comparably, the constitutively filamentous *Δtup1* mutant strain induced neutrophil ROS in a similar fashion as pre-grown wild-type hyphae, which was even true at a high DM amount up to 40 µg (Fig. 2B). Only at DM infection doses below 5 µg, the *Δtup1* mutant induced a slightly higher measurable ROS signal than wild-type hyphae.

Taken together, our findings with morphotype-locked strains indicate that the initial *C. albicans* morphotype shapes the neutrophil ROS response. Changes of morphology during the assay have most probably only minor effects on the induction of ROS.

### Killed versus Live *C. albicans*-induced ROS in Neutrophils

We next assessed the neutrophil ROS response towards dead *C. albicans*, which are often used in infection experiments. We infected neutrophils with different doses of heat-killed, denatured or thimerosal-killed, structurally preserved *C. albicans* [33] and measured ROS production as described. ROS production by neutrophils upon infection with heat-killed *C. albicans* was dependent on the infection dose. Low DM of heat-killed *C. albicans* (<5 µg per 10^5^ neutrophils) induced negligible amounts of ROS compared to live *C. albicans*. This is true for both heat-killed yeasts (Fig. 2C) and hyphae (Fig. 2D). Neutrophil ROS increased in a dose-dependent manner at doses higher than 5 µg heat-killed *C. albicans* yeasts, without reaching a maximum up to 40 µg DM (Fig. 2C). However, the total amount of ROS production ranged below the maximum value reached for live *C. albicans* yeasts. Above a dose of 15 µg, neutrophil ROS production induced by heat-killed yeasts exceeded the declining ROS from neutrophils stimulated with viable yeasts (Fig. 2C). In comparison to yeasts, dead hyphae did not evoke any measurable ROS up to doses of 5 µg (Fig. 2D). Above 5 µg, the neutrophil ROS production increased rapidly until exceeding those values induced by viable *C. albicans* hyphae at doses higher than 20 µg (Fig. 2D).

Interestingly, infection of neutrophils with thimerosal-killed, *C. albicans* provoked only very low ROS production. This was the case for both morphotypes (Fig. 2C and D) indicating that thimerosal-killed *C. albicans* efficiently scavenge ROS, rather than actively suppress the production.

### *C. albicans* does not Suppress NADPH Oxidase but Detoxifies Neutrophil ROS

To elucidate the mechanisms governing the distinct ROS responses in neutrophils induced by the two *C. albicans* morphotypes studied, a “spiking assay” was performed. For this purpose, neutrophils were infected with *C. albicans* yeasts or hyphae. After 30 min, a second infection dose (spike) of *C. albicans* was added in order to elucidate whether neutrophils are still able to respond to this second, spiking infection dose.

**Figure 3:**
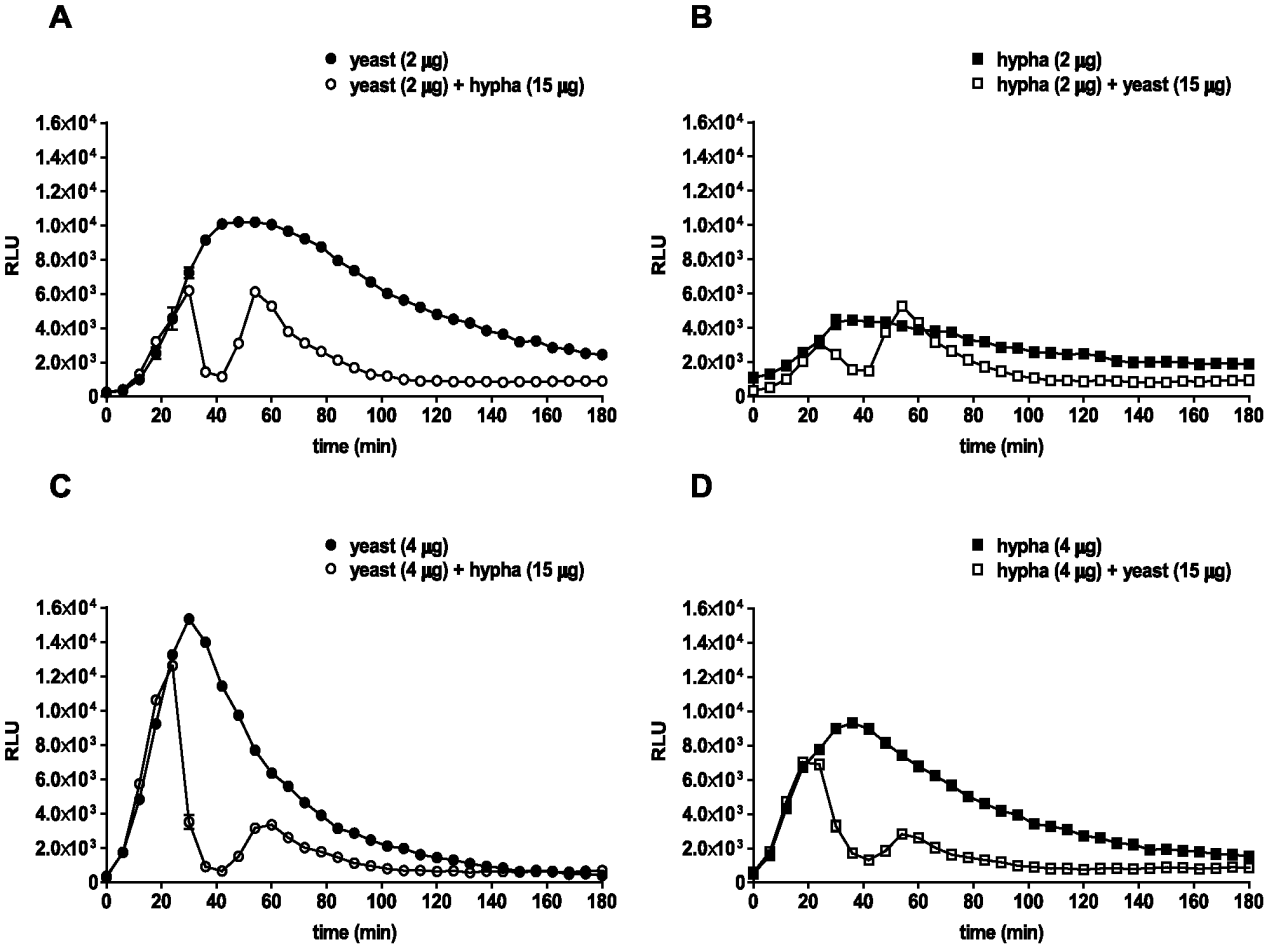
*C. albicans* removes ROS in primary and induces additional ROS in secondary infections of neutrophils. Spiking ROS assay I: ROS production over time is shown for total of 3 h as relative light units (RLUs). For each sample, 10^5^ neutrophils were infected, either with 2 µg (A) and 4 µg (C) *C. albicans* yeasts (closed circles) and after 30 min re-infected with 15 µg *C. albicans* hyphae (open circles) or with 2 µg (B) and 4µg (D) *C. albicans* hyphae (closed squares) and re-infected with 15 µg *C. albicans* yeasts (open squares). A representative experiment out of three independent experiments from three different donors is shown. Data are presented as means of three technical replicates ±SD. Masses to MOI conversion for 10^5^ neutrophils: (Yeasts) 2 µg≙0.8, 4 µg≙1.6, 15 µg≙6.2; (hyphae) 2 µg≙0.3, 4 µg≙0.7, 15 µg≙2.6.

**Figure 4:**
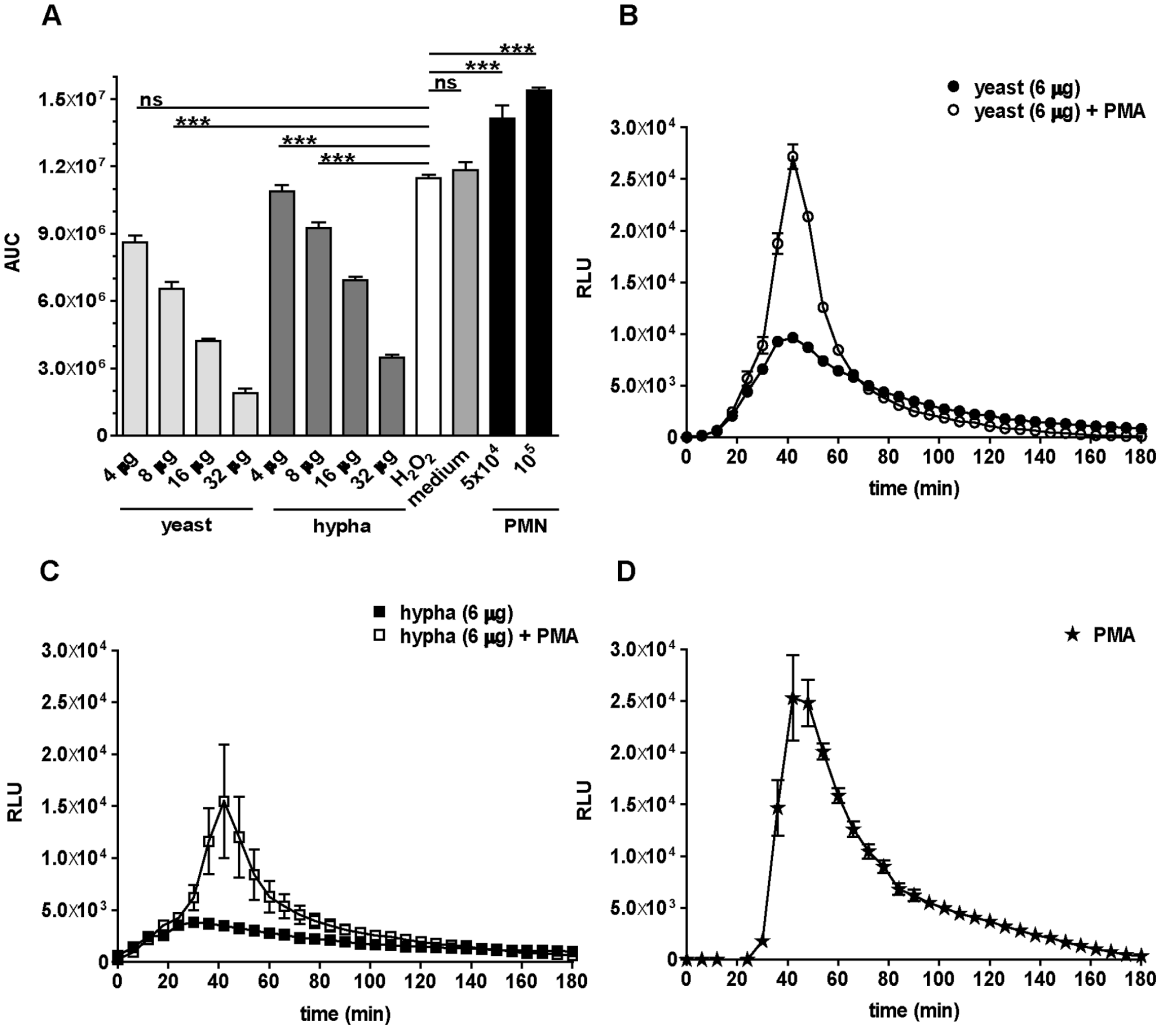
*C. albicans* directly detoxifies ROS, but cannot block PMA-induced neutrophil ROS. ROS scavenging: Different masses of *C. albicans* yeasts and hyphae, RPMI medium and unstimulated neutrophils were added to hydrogen peroxide. Each column represents the corresponding AUC from RLUs measured by luminol (A). A representative experiment out of three independent experiments from three different donors is shown. Data are presented as means of three technical replicates ±SD (NS: P>0.05 and ***P≤0.001). Spiking ROS assay II: ROS production over time is shown for a total of 3 h as relative light units (RLUs). For each sample, 10^5^ neutrophils were infected with 6 µg *C. albicans* yeasts (closed circles, B) or hyphae (closed squares, C) and after 30 min PMA was added. Additionally, neutrophils were stimulated with 100 nM PMA only (stars, D). A representative experiment out of three independent experiments from three different donors is shown. Data are presented as means of three technical replicates ±SD. Masses to MOI conversion for 10^5^ neutrophils: (Yeasts) 4 µg≙1.6, 6 µg≙2.5, 8 µg≙3.3, 16 µg≙6.6, 32 µg≙13.1; (hyphae) 4 µg≙0.7, 6 µg≙1, 8 µg≙1.4, 16 µg≙2.7, 32 µg≙5.4.

In response to the first infection, measurable neutrophil ROS production increased steadily until reaching a maximum between 30–50 min p.i, after which the signal continuously declined to negligible amounts during the course of 3 h. Interestingly, in these spiking assays, in which either yeasts (Fig. 3A and C) or hyphae (Fig. 3B and D) were added to the first infection, we observed two distinct neutrophil ROS peaks. The addition of a second infection dose at 15 µg of fungal pathogen resulted in an immediate decline of the first ROS peak. After approximately 40 min however, the neutrophil ROS signal increased again reaching a second peak after about 60 min. This second neutrophil ROS peak demonstrates that the ROS generation machinery is not actively suppressed by live *C. albicans*. Instead, the fungus efficiently removed neutrophil ROS. The spiking experiments were performed at several primary amounts of both *C. albicans* morphotypes. At the primary infection dose of 2 µg (Fig. 3A–B) the secondary ROS peak was more apparent than the secondary peak at a higher primary infection dose (Fig. 3C–D). This might be due to a lower detoxification capacity of the lower primary infection dose. The potent drop of the first peak to basal levels indicates active ROS detoxification by both morphotypes in a dose-dependent manner. In order to dissect the contributions of individual assay components to the detoxification of ROS, we performed an experiment with an exogenous source of ROS. We added different amounts of *C. albicans* yeasts, hyphae, medium or neutrophils to H_2_O_2_. Remaining peroxide was quantified using luminol. We observed a dose-dependent removal of H_2_O_2_ by live *C. albicans* (Fig. 4A). The highest mass of yeasts applied resulted in the removal of more than 80% of H_2_O_2_, whereas RPMI medium alone had no effect. The presence of neutrophils slightly increased measurable peroxides and thus did not scavenge H_2_O_2_. Thus, removal of neutrophil ROS is largely mediated by *C. albicans* rather than by media components, or neutrophils. Additionally, this experiment revealed that wild-type *C. albicans* yeasts remove ROS slightly more efficient than hyphae (Fig. 4A).

Phorbol-12-myristate-13-acetate (PMA) is a strong activator of PKC, a main regulator of the oxidative burst machinery in neutrophils. To confirm that the ability of neutrophils to produce ROS was not suppressed downstream of PKC by *C. albicans*, we infected neutrophils either with yeasts (Fig. 4B) or hyphae (Fig. 4C) and spiked with PMA 30 min thereafter (Fig. 4B–C). ROS generation in neutrophils was monitored and increased directly upon addition of PMA (Fig. 4B–D) indicating that *C. albicans* does detoxify ROS, rather than actively suppressing the neutrophil ROS machinery.

### *C. albicans* Detoxifies Neutrophil ROS using Extracellular SOD Proteins

**Figure 5:**
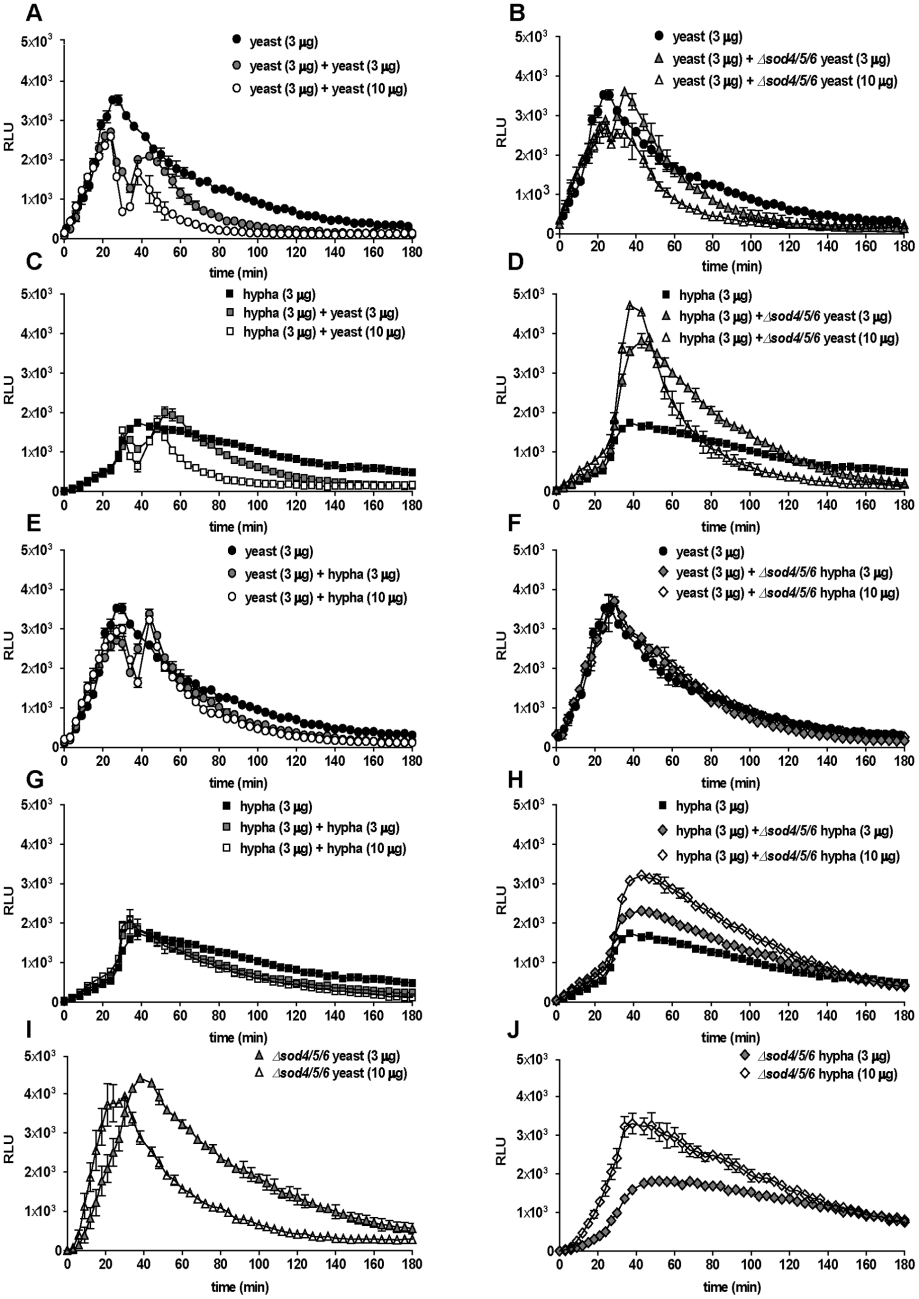
Detoxification of neutrophil ROS is severely impaired in *C. albicans Δsod4/5/6* mutant. Spiking ROS assay III: ROS production over time is shown for a total of 3 h as relative light units (RLUs). For each sample, 10^5^ neutrophils were infected. The first infection dose were 3 µg of wild-type *C. albicans* yeasts (black circles, A-B and E-F) or 3 µg of wild-type *C. albicans* hyphae (black squares, C-D and G-H); 30 min thereafter, the neutrophils were re-infected with 3 µg (gray symbols) or 10 µg (white symbols) wild-type *C. albicans* yeasts (A and C), *Δsod4/5/6* mutant strain yeasts (B and D), wild-type hyphae (E and G) or *Δsod4/5/6* mutant strain hyphae (F and H). Additionally, neutrophils were solely infected with 3 µg (gray triangles) and 10 µg (white triangles) of *Δsod4/5/6* yeasts (I), or with 3 µg (gray rhombuses) and 10 µg (white rhombuses) *Δsod4/5/6* hyphae (J). A representative experiment out of three independent experiments from three different donors is shown. Data are presented as means of three technical replicates ±SD. Masses to MOI conversion for 10^5^ neutrophils: (Yeasts) 3 µg≙1.2, 10 µg≙4.1; (hyphae) 3 µg≙0.5, 10 µg≙1.7.

*S*urface-localized *SOD* proteins from *C. albicans* detoxify ROS generated by mouse macrophages, dendritic cells and neutrophils. [18], [20]. We performed a spiking assay, in which neutrophils were infected with wild-type *C. albicans* yeasts or hyphae and after 30 min spiked with either wild-type (Fig. 5A, C, E and G) or *Δsod4/5/6* triple-knockout strain (Fig. 5B, D, F and H). Both morphotypes of *Δsod4/5/6* mutant strains were significantly impaired in scavenging neutrophil ROS. Spiking of wild-type *C. albicans* yeasts with *Δsod4/5/6* yeasts resulted in a reduction of the scavenging effect compared to spiking with wild type (Fig. 5A and B). Spiking of wild-type *C. albicans* hyphae with *Δsod4/5/6* yeasts even resulted in an increased total ROS signal (Fig. 5C and D). Using *Δsod4/5/6* hyphae to spike wild-type *C. albicans* yeasts abrogated ROS scavenging (Fig. 5E and F) and also led to an overall increased ROS signal for wild-type *C. albicans* hyphae (Fig. 5G and H). The *Δsod4/5/6* mutant strain alone triggered more ROS in neutrophils (Fig. 5I and J) consistent with a previous report [20]. Moreover, *C. albicans* wild-type yeasts seem to be more effective in scavenging ROS compared to hyphae, as in spiking experiments hyphae consistently removed less ROS than their yeast counterparts (Fig. 5A and E as well as C and G). Together, this confirms that the capacity of *C. albicans* to reduce neutrophil ROS most probably stems from detoxification, rather than from active suppression of ROS production. *SOD5* is the most important of the extracellular SODs during the interaction with phagocytes [20]. To confirm this we compared the triple mutant strain *Δsod4/5/*6 to a *Δsod5* single knockout mutant and the corresponding revertant strain *Δsod5/SOD5* [18]. We stimulated neutrophils with PMA and added 10 µg *Δsod4/5/6, Δsod5, Δsod5/SOD5* or wild-type hyphae 20 min thereafter. ROS were measured and the AUCs calculated as described above (Fig. S4A). Whereas the triple and single mutants did not scavenge any neutrophil ROS, the *Δsod5/SOD5* revertant strain showed similar ROS removal capacity as the wild type. This corroborates that Sod5p is the most important SOD protein for the removal of neutrophil ROS.

**Figure 6:**
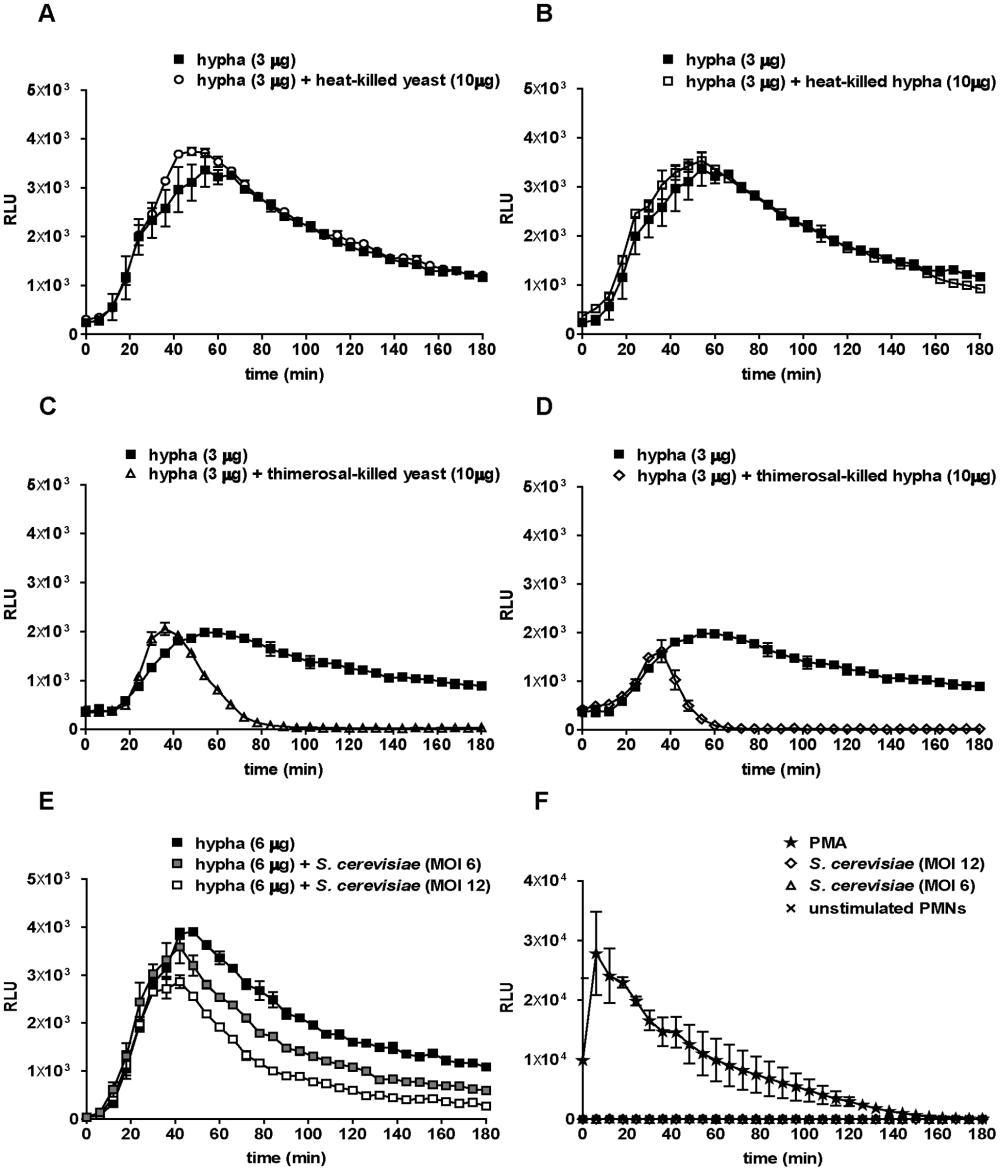
Not heat-killed, but thimerosal-killed *C. albicans* and live *S. cerevisiae* detoxify neutrophil ROS. Spiking ROS assay IV: ROS production over time is shown for a total of 3 h as relative light units (RLUs). For each sample, 10^5^ neutrophils were infected with a first infection dose of 3 µg of *C. albicans* wild-type hyphae (closed squares, A-D); 30 min thereafter, the cells were re-infected with 10 µg of *C. albicans* heat-killed yeasts (open circles, A) or of heat-killed hyphae (open squares, B), with 10 µg of thimerosal-killed yeasts (open triangles, C) or of thimerosal-killed hyphae (open rhombuses, D). In (E), neutrophils were infected with a first infection dose of 6 µg of *C. albicans* wild-type hyphae (closed squares) and 30 min thereafter re-infected with *S. cerevisiae* at MOI 12 (gray squares) or MOI 6 (open squares). In (F), neutrophils were stimulated with PMA (100 nM, star), different MOIs of *S. cerevisiae* (rhombuses and triangles) or remained unstimulated (cross). A representative experiment out of three independent experiments from three different donors is shown. Data are presented as means of three technical replicates ±SD. Masses to MOI conversion for 10^5^ neutrophils: (Yeasts) 10 µg≙4.1; (hyphae) 3 µg≙0.5, 6 µg≙1, 10 µg≙1.7.

To confirm that the detoxification capacity of *C. albicans* shaped the neutrophil ROS response, we used heat-killed and thimerosal-killed *C. albicans.* Live wild-type *C. albicans* hyphae were spiked with either form of killed *C. albicans* (Fig. 6). As expected, no detoxification of neutrophil ROS was observed when neutrophils were spiked with heat-killed *C. albicans* (Fig. 6A–B). In contrast, although thimerosal-treated *C. albicans* were completely deactivated as confirmed by plating on growth media (data not shown), the scavenging effect was still evident (Fig. 6C–D). This detoxifying effect was observed with spiking of both growth forms of *C. albicans*. Thus, deactivation of *C. albicans* by thimerosal preserved the activity of ROS-degrading enzymes. However, a second ROS peak was absent which might be due to a less pronounced induction of neutrophil ROS by dead *C. albicans* in spite of effective ROS scavenging. Moreover, these findings explain the low neutrophil ROS response towards thimerosal-killed *C. albicans* yeasts and hyphae. In summary, both morphotypes detoxified neutrophil ROS effectively and the more potent scavenging effect from *C. albicans* yeasts leads to less measurable neutrophil ROS at high infection doses. Residual thimerosal leaking from killed *C. albicans* might inhibit neutrophils resulting in low ROS responses. Therefore, we infected human neutrophils with different amounts of thimerosal-killed hyphae and plotted ROS signals over time (Fig. S4B). We observed low ROS production, which was dependent on the dose of thimerosal-killed hyphae, demonstrating that inhibition of neutrophils by residual thimerosal did not occur (Fig. S4B). Two h p.i. we added PMA to each sample. The previously infected neutrophils responded to the phorbol ester with a robust oxidative burst (Fig. S4B). The height of the peak however declined with increasing amounts of thimerosal-killed hyphae, indicating that these hyphae indeed scavenged neutrophil ROS efficiently.

Finally, we aimed to elucidate whether the ROS scavenging effect was unique for *C. albicans*. For this purpose, we used *S. cerevisiae* in spiking experiments. We infected human neutrophils with wild-type *C. albicans* yeasts (data not shown) and hyphae (Fig. 6E) and spiked with different amounts of *S. cerevisiae* at MOI 12 and MOI 6 (Fig. 6E). The number of *S. cerevisiae* yeast cells was used instead of DM since the correlation to DM for *S. cerevisiae* was not relevant for this study. Notably, *S. cerevisiae* UMY2067 wild-type strains did not evoke any measurable ROS in neutrophils at different MOIs in comparison to PMA-induced controls (Fig. 6F). However, UMY2067 nevertheless efficiently detoxified neutrophil ROS produced upon *C. albicans* stimulation (Fig. 6E). Again, a secondary ROS peak was absent in consistency with the notion that UMY2067 itself did not induce neutrophil ROS production.

### Neutrophil Elicit Specific IL-8 Secretion Patterns Towards *C. albicans* Morphotypes

**Figure 7:**
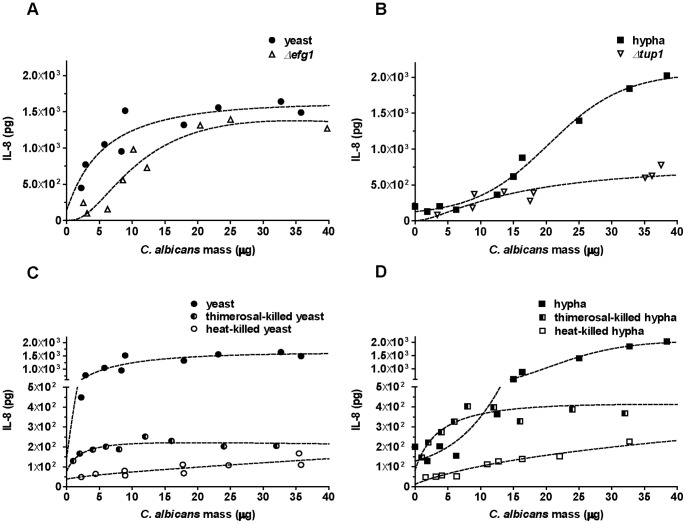
Neutrophil IL-8 response pattern depends on amount, morphotype and viability of *C. albicans*. Neutrophil IL-8 secretion was measured by ELISA. For each sample, 10^5^ neutrophils were infected with *C. albicans*. Each data point represents the total amount of IL-8 secreted by neutrophils in the course of 6 h. To compare C. *albicans* wild-type to morphotype-locked mutants neutrophils were infected with wild-type yeasts (closed circles, A), the *Δefg1* mutant (open triangles, A), wild-type hyphae (closed squares, B) or the *Δtup1* mutant (open inverted triangles, B). Neutrophils were infected with C. *albicans* wild-type yeasts which were alive (closed circles, C), thimerosal-killed (half-closed circles, C) or heat-killed (open circles, C) or were infected with C. *albicans* wild-type hyphae which were alive (closed squares, D), thimerosal-killed (half-closed squares, D) or heat-killed (open squares, D). The dashed line indicates the best-fit which was obtained from three individual assays from three different donors. Masses to MOI conversion for 10^5^ neutrophils: (Yeasts) 1 µg≙0.4, 5 µg≙2.1, 10 µg≙4.1, 20 µg≙8.2, 30 µg≙12.3; (hyphae) 1 µg≙0.2, 5 µg≙0.9, 10 µg≙1.7, 20 µg≙3.4, 30 µg≙5.1.

Relying on *C. albicans* mass correlation instead of MOI provided new insight in specific neutrophil ROS responses. The secretion of the pro-inflammatory chemokine IL-8 is an important response of neutrophils towards different stimuli [16]. As demonstrated above the IL-8 response pattern of neutrophils towards live *C. albicans* yeasts or hyphae resembled the ROS response pattern (Fig. 1B). At lower infection doses (5–20 µg), yeasts induced higher release of IL-8 than hyphae, whereas hyphae trigger more IL-8 secretion above 25 µg infection dose (Fig. 1B). The neutophil IL-8 responses to mutant strains arrested in each growth form were also studied (Fig. 7A–B). The yeast-locked *Δefg1* mutant triggered very similar, albeit slightly lower IL-8 secretion in neutrophils than wild-type *C. albicans* (Fig. 7A). At infection doses below 10 µg, the neutrophil IL-8 release triggered by both wild-type and yeast-locked *C. albicans* was higher than release induced by filamentous *C. albicans* (Fig. 7B). The constitutively filamentous *Δtup1* mutant however, could not serve as control in these experiments, since it induced considerably less IL-8 secretion by neutrophils than wild-type hyphae, which agrees well with a previous report [12]. Given that wild-type hyphae continue growing as hyphae under the conditions used, these results nonetheless indicate that IL-8 secretion of neutrophils upon *C. albicans* infection depends on both amounts and morphotypes.

Analogously to the ROS response, we also tested IL-8 release upon infection with heat-killed and thimerosal-killed *C. albicans*. The pattern was again comparable to the ROS response (Fig. 7C and D). IL-8 in the supernatant of yeast- and hypha-infected neutrophils was overall significantly lower as compared to stimulation with viable *C. albicans*. Thimerosal-killed *C. albicans* induced IL-8 secretion in a dose-dependent manner only at infection doses below 5µg for both yeasts and hyphae (Fig. 7C and D). At higher infection doses, the IL-8 release from neutrophils remained fairly constant at approximately 200 pg/ml for yeast and 400 pg/ml for hypha infection. The IL-8 response to heat-killed *C. albicans* was dose-dependent throughout the whole DM range used, but lower compared to thimerosal-killed *Candida* (10–100 pg/ml for both yeast and hypha infection). This indicates that human neutrophils recognize viable *C. albicans* differently than dead *C. albicans* and launch a specific IL-8 response. The mechanism behind this differential recognition remains to be solved.

## Discussion

Immune cells have been suggested to shape pathogen-induced responses meticulously according to the morphology and amount of microbes encountered to ensure a balanced and appropriate counter attack [15]. To investigate host cell and microbe interactions with a controlled ratio of host and pathogen, MOIs are a widely accepted standard measure. While this approach seems appropriate for many bacteria that divide exclusively by binary fission, it is less applicable for polymorphic microorganisms, such as *C. albicans*. The polymorphic nature of *C. albicans* renders comparative studies of immune cell responses towards different growth forms challenging. During yeast growth, an increase in cell mass, cell surface and MA is accompanied by a rise in cell number, whereas filamentous growth does not augment the number of discriminable cellular entities. To solve this, we chose a DM-based approach for the accurate determination of amounts of *C. albicans* morphotypes used in neutrophil infection experiments. We showed that DM, as opposed to MOI, is directly correlated to the cell surface area of the microbial entity. Using an array of equations to directly correlate DM to MA and OD we could infect human neutrophils with equal amounts and thus a comparable cell surface area of *C. albicans,* irrespective of morphotype.

Our findings agree with previous data describing that 1 µg DM of *C. albicans* yeasts correspond to 4×10^4^ cells [18]. Furthermore, we also evaluated the cell surface area of yeast and hyphal cells. Interestingly, the ratio of cell surface area per µg DM of yeasts versus hyphae remained constant, whereas the ratio per cell number considerably increased over time (Table 1). This demonstrates that the cell surface area indeed directly correlates to the DM. Therefore, the DM approach allows infection of neutrophils with equal microbial surface areas, which constitute the interface between microbial and host cell.

With these established tools in hand we set the stage to explore differential responses of human neutrophils towards *C. albicans* yeasts and hyphae cultured under standard laboratory conditions. We provide insight into neutrophil ROS responses over a wide range of amounts of *C. albicans* yeast and hyphae as detailed as possible. Remarkably, we found that low infection doses of live *C. albicans* yeasts induce more ROS than their hyphal counterparts. This finding is unexpected, since hyphae, the invaders of tissue, are described as immune-activating [12], [34]. Human neutrophils have been shown to migrate faster towards, and to preferentially engulf, hyphal *C. albicans* [12], [34]. However, our findings demonstrate that neutrophils launch a strong ROS response upon infection with *C. albicans* yeasts. Since a yeast-locked *Δefg1* mutant strain also induced significantly higher ROS levels than hyphae at doses below 5 µg, it is very unlikely that high ROS production at low amounts of yeasts solely result from germ tube formation during the assay period. This was true even though the overall ROS induction by *Δefg1* was lower than by wild-type *C. albicans*. In contrast, amounts above 15 µg of *C. albicans* hyphae evoked a higher neutrophil ROS response than the equivalent amount of yeasts and the constitutively filamentous *Δtup1* mutant strain induced a very similar neutrophil ROS pattern as compared to wild-type hyphae. Together, our data indicates that the initial form of infection shapes the neutrophil ROS response. However, due to the fact that filamentous *C. albicans* tend to clump, available cell surface areas might be restricted, resulting in an overall lower immune cell response than that induced by yeast-form *C. albicans*, which is less prone to aggregation. It is important to note that the morphologic form of *C. albicans* is not solely responsible for determining immune responses. Other regulatory factors acting independent of morphology are also relevant. For instance, macrophages released less IL-1β upon infection with transcription factor-deleted *Δupc2* strain than wild-type *C. albicans*, although this mutant was competent in forming hyphae [35]. Furthermore, in this study we induced yeast growth in YPD at 30°C and hyphal growth in RPMI at 37°C and thus the two morphotypes were grown in different media and under different temperatures. Environmental cues and media components influence the biology of *C. albicans* to a large extent. For instance, the cell wall composition changes under growth in different media [36] and thus, the type of growth media could contribute to altered immune responses towards *C. albicans*. Therefore, we cannot fully exclude the possibility that growth specific-alterations in *C. albicans* could contribute to specific patterns of neutrophil response. Importantly however, we cultivated the *Δtup1* strain in YPD at 30°C, since it grows constitutively as a filamentous form and the ROS response of neutrophils to this mutant was very similar to the response to wild-type hyphae. This is indeed an indication that the morphology, rather than media-specific or mutant-specific effects determined the neutrophil response under these conditions. Thus, our study serves as a starting point to more accurately investigate differences in morphotype-dependent immune responses towards *C. albicans* growth forms cultured under standard laboratory conditions.

In agreement with an earlier study [30] amounts above 10 µg of *C. albicans* yeast cells led to a decline of measurable phagocyte ROS (Fig. 1). Another report suggested that PMA-stimulated ROS produced in dendritic cells is inhibited by *C. albicans* [37]. However, we demonstrate here that in human neutrophils the decline was not due to active suppression of ROS production, but originated from efficient ROS detoxification. Several lines of evidence support this notion: (i) Secondary infection with large amounts of *C. albicans* reduced measurable ROS immediately, but resulted in a secondary ROS peak indicating that no active ROS blockage had occurred; (ii) the multiple SOD mutant strain used for secondary infection could not decrease measurable ROS to the same extent, but rather resulted in increased total amounts of ROS; (iii) thimerosal-killed, structurally preserved *C. albicans* as well as live fungi reduced the levels of measured ROS immediately, whereas heat-killed, denatured *C. albicans* left measurable ROS levels unchanged; and (iv) secondary infection with non-pathogenic *S. cerevisiae* also evoked a decline in ROS, but did not result in a second ROS peak. Extracellular SODs in *C. albicans* were previously reported to play a role in avoiding extracellular oxidative stress [22], [38]. These enzymes were shown to remove ROS from macrophages and dendritic cells [18]. A previous study identified Sod5p as the major antioxidant defense of *C. albicans* against human neutrophils [20], which is in good agreement with our findings. However, to our knowledge this is the first description of spiking experiments to analyze ROS responses of human neutrophils. SODs are not the only ROS detoxifying enzymes of *C. albicans*, nonetheless our spiking experiments demonstrate that during interaction with human neutrophils a significant proportion of phagocyte ROS is removed via this route (Fig. 5). Moreover, we investigated differences in stimulation of human neutrophils by either live or dead *C. albicans*. This is of importance, since many studies have used dead *C. albicans* to assess immune cell responses in order to avoid mixed growth morphotypes and overgrowth [39]. We found that low doses of live *C. albicans* induce significantly more ROS in neutrophils than comparable doses of heat-killed *C. albicans* (Fig. 2). In contrast, at high infection doses, killed *C. albicans*, in particular the hyphal form, induced higher ROS amounts than live fungi. However, this finding is complicated by the fact that high amounts of live *C. albicans* efficiently detoxify ROS. We additionally showed that heat-killed *C. albicans* are unable to detoxify neutrophil ROS (Fig. 6A and B), whereas thimerosal-killed *C. albicans* were unexpectedly efficient in removing ROS (Fig. 6C and D). We conclude that induction of less ROS and efficient detoxification accumulate in thimerosal-killed fungi (Fig. S4B) and that human neutrophils might sense viability of *C. albicans* with a hitherto unknown mechanism. As neutrophils specifically detect secreted pH-regulated antigen 1 protein (Pra1p) via integrin β2 signalling [40], this cell surface protein could be a candidate for the discrimination between live and dead *C. albicans* cells. However, yeast-form cells should not release this protein and thus further studies are required to determine the differential recognition of live and dead *C. albicans* by human neutrophils.

Interestingly, similarities in the IL-8 and ROS response patterns of human neutrophils could be observed upon stimulation with live *C. albicans*. At low doses, yeast-form *C. albicans* provoked more IL-8 secretion than hyphae, whereas at doses above 25 µg, hyphae stimulated higher amounts of released IL-8. This is surprising, since we measured IL-8 concentrations in the supernatant 6 h p.i. At this time point large hyphae can be formed under the assay conditions. However, it cannot be elucidated entirely to which extent the initial form of infection determines the IL-8 response. The yeast-locked *Δefg1* mutant did induce a similar dose-dependent pattern, but to a lower extend as compared to the wild-type *C. albicans* strain. Nevertheless, the IL-8 concentration in the supernatant was higher upon stimulation with the *Δefg1* mutant than with wild-type hyphae at doses below 15 µg, indicating that a higher IL-8 release from neutrophils did not solely stem from recognition of germination (Fig. 7). The constitutively filamentous *Δtup1* mutant however, did not induce considerable amounts of IL-8 release, which is in accordance to a previous report [12]. The deficiency of the morphotype-locked *Δtup1* strain to induce an IL-8 response in neutrophils again illustrates that the use of such strains is limited. These mutants are generally not exclusively unable to change morphology, but also display additional deviations from wild-type strains, such as the expression of different levels of detoxifying enzymes or immune modulatory enzymes or epitopes and can thereby obscure the correct interpretation of results. Dead *C. albicans* in contrast induced consistently less IL-8 release from human neutrophils than live *C. albicans*, whereas dead hyphae induced more IL-8 release than dead yeasts. Heat-killed and structurally denatured fungi elicited the overall lowest response. Only at doses below 10 µg, thimerosal-killed hyphae stimulated a similar or slightly higher neutrophil IL-8 secretion than viable counterparts. This again suggests that neutrophils are able to sense viability of *C. albicans*.

In summary, we present a new approach by which to compare immune cell responses towards different morphologies of the same microbe, exemplified for neutrophil ROS production and IL-8 secretion upon infection with different amounts and morphotypes of *C. albicans*. We have shown that neutrophil ROS production and IL-8 secretion upon *C. albicans* infection is not constantly higher or lower for one morphotype, but that this largely depends on the dose of infection. Viable *Candida* cells result generally in higher neutrophil responses than dead cells. While *C. albicans* might be able to actively suppress ROS production of certain immune cells, the fungus is unable to do so in human neutrophils. Instead, *C. albicans* efficiently detoxifies neutrophil ROS, in part by the use of extracellular SODs. In conclusion, our approach to use dry mass as denominator for equal amounts of different microbial morphotypes can serve as a blueprint for other studies investigating the interactions of different strains of *C. albicans* or other polymorphic fungal species with immune cells to give new insight into meticulous regulation of immune responses.

## Supporting Information

Figure S1 **Determination of** ***C. albicans*** **cell surface area.** The cell surface area was calculated by measuring the dimensions of immuno-stained *C. albicans* (anti-*Candida* antibody). Illustrated here are yeast at 1 h (A) and hyphae at 2 (B), 3 (C) and 4 h (D) after induction, respectively. The overall cell surface area was calculated by using the ellipsoid formula for budding yeasts and the cylinder formula for hyphae. Confocal microscopy (Nikon C1 confocal microscope, NIS-Elements AR, version 3.2.0) was applied and z-stacks were obtained from an average of 50 images to measure the volume of *C. albicans* cells at different time points.
(TIF)

Figure S2 **DM measurement of wild-type and mutant** ***C. albicans*** **strains.** Aliquots of liquid culture at different time point were concentrated on paper filters and subsequently dried and weighed. DM was plotted versus time for wild-type and mutant strains. The wild-type yeast *C. albicans* (R^2^ = 0.99) (A) and the *Δefg1* mutant strain (C) cultured in YPD medium at 30°C (R^2^ = 0.93). Wild-type hyphae *C. albicans* (B) cultured in RPMI medium at 37°C (R^2^ = 0.85) and the *Δtup1* mutant (D) cultured in YPD medium at 30°C (R^2^ = 0.93). The line indicates the best-fit obtained from three biological replicates.
(TIF)

Figure S3 **Measuring OD, DM and MA of wild-type and morphotype-locked** ***C. albicans*** **mutants.** OD and XTT values versus time, as well as DM versus OD and XTT values, were plotted for wild-type yeast (A, D, G and J) and the *Δefg1* mutant strain (B, E, H and K). For the filamentous growth forms XTT values versus time and DM versus XTT values were plotted for wild-type hyphae (C and F) and the *Δtup1* mutant strain (I and L). The line indicates the best-fit obtained from three representative biological replicates.
(TIF)

Figure S4 **Live and thimerosal-killed** ***C. albicans*** **detoxify neutrophil ROS.** Spiking ROS assay: Each column represents AUC corresponding to the total ROS generated by neutrophils over 180 min. Neutrophils (10^5^) were stimulated with PMA (100 nM). After 20 min neutrophils were spiked with 10 µg *C. albicans Δsod4/5/6*, *Δsod5, Δsod5:SOD5,* wild-type hypha (A). Relative Light Units (RLUs) correspond to neutrophil ROS in a luminol-based chemiluminescence assay. For each sample 10^5^ neutrophils were infected with different masses of thimerosal-killed *C. albicans*; after 120 min, the neutrophils were spiked with 100 nM PMA (B). Data are presented as means of three technical replicates ±SD (NS: P>0.05, **P≤0.01 and ***P≤0.001). Masses to MOI conversion for 10^5^ neutrophils: (Hyphae) 1 µg≙0.2, 8 µg≙1.4, 16 µg≙2.7, 32 µg≙5.4.
(TIF)

## References

[1] Odds FC (1987) Candida infections: an overview. Crit Rev Microbiol 15: 1–5. 10.3109/10408418709104444 3319417

[2] Hajjeh RA, Sofair AN, Harrison LH, Lyon GM, Arthington-Skaggs BA, et al (2004) Incidence of bloodstream infections due to Candida species and in vitro susceptibilities of isolates collected from 1998 to 2000 in a population-based active surveillance program. J Clin Microbiol 42: 1519–1527. 15070998 10.1128/JCM.42.4.1519-1527.2004 PMC387610

[3] Gow NA, Gooday GW (1987) Cytological aspects of dimorphism in Candida albicans. Crit Rev Microbiol 15: 73–78. 3319423 10.3109/10408418709104449

[4] Whiteway M, Bachewich C (2007) Morphogenesis in Candida albicans. Annu Rev Microbiol 61: 529–553. 17506678 10.1146/annurev.micro.61.080706.093341 PMC4452225

[5] Cottier F, Muhlschlegel FA (2009) Sensing the environment: response of Candida albicans to the X factor. FEMS Microbiol Lett 295: 1–9. 19473245 10.1111/j.1574-6968.2009.01564.x

[6] Calderone RA, Fonzi WA (2001) Virulence factors of Candida albicans. Trends Microbiol 9: 327–335. 11435107 10.1016/s0966-842x(01)02094-7

[7] Sudbery P, Gow N, Berman J (2004) The distinct morphogenic states of Candida albicans. Trends Microbiol 12: 317–324. 15223059 10.1016/j.tim.2004.05.008

[8] Stoldt VR, Sonneborn A, Leuker CE, Ernst JF (1997) Efg1p, an essential regulator of morphogenesis of the human pathogen Candida albicans, is a member of a conserved class of bHLH proteins regulating morphogenetic processes in fungi. EMBO J 16: 1982–1991. 9155024 10.1093/emboj/16.8.1982 PMC1169801

[9] Braun BR, Johnson AD (1997) Control of filament formation in Candida albicans by the transcriptional repressor TUP1. Science 277: 105–109. 9204892 10.1126/science.277.5322.105

[10] Young G (1958) The process of invasion and the persistence of Candida albicans injected intraperitoneally into mice. J Infect Dis 102: 114–120. 13525770 10.1093/infdis/102.2.114

[11] d’Ostiani CF, Del Sero G, Bacci A, Montagnoli C, Spreca A, et al (2000) Dendritic cells discriminate between yeasts and hyphae of the fungus Candida albicans. Implications for initiation of T helper cell immunity in vitro and in vivo. J Exp Med 191: 1661–1674. 10811860 10.1084/jem.191.10.1661 PMC2193147

[12] Wozniok I, Hornbach A, Schmitt C, Frosch M, Einsele H, et al (2008) Induction of ERK-kinase signalling triggers morphotype-specific killing of Candida albicans filaments by human neutrophils. Cell Microbiol 10: 807–820. 18034864 10.1111/j.1462-5822.2007.01086.x

[13] Moyes DL, Runglall M, Murciano C, Shen C, Nayar D, et al (2010) A biphasic innate immune MAPK response discriminates between the yeast and hyphal forms of Candida albicans in epithelial cells. Cell Host Microbe 8: 225–235. 20833374 10.1016/j.chom.2010.08.002 PMC2991069

[14] Netea MG, Kullberg BJ (2010) Epithelial sensing of fungal invasion. Cell Host Microbe 8: 219–220. 20833371 10.1016/j.chom.2010.08.008

[15] Netea MG, Gow NA, Munro CA, Bates S, Collins C, et al (2006) Immune sensing of Candida albicans requires cooperative recognition of mannans and glucans by lectin and Toll-like receptors. J Clin Invest 116: 1642–1650. 16710478 10.1172/JCI27114 PMC1462942

[16] Miller MD, Krangel MS (1992) Biology and biochemistry of the chemokines: a family of chemotactic and inflammatory cytokines. Crit Rev Immunol 12: 17–46. 1418604

[17] Dinauer MC (1993) The respiratory burst oxidase and the molecular genetics of chronic granulomatous disease. Crit Rev Clin Lab Sci 30: 329–369. 8110374 10.3109/10408369309082591

[18] Frohner IE, Bourgeois C, Yatsyk K, Majer O, Kuchler K (2009) Candida albicans cell surface superoxide dismutases degrade host-derived reactive oxygen species to escape innate immune surveillance. Mol Microbiol 71: 240–252. 19019164 10.1111/j.1365-2958.2008.06528.x PMC2713856

[19] Chauhan N, Latge JP, Calderone R (2006) Signalling and oxidant adaptation in Candida albicans and Aspergillus fumigatus. Nat Rev Microbiol 4: 435–444. 16710324 10.1038/nrmicro1426

[20] Miramon P, Dunker C, Windecker H, Bohovych IM, Brown AJ, et al (2012) Cellular responses of Candida albicans to phagocytosis and the extracellular activities of neutrophils are critical to counteract carbohydrate starvation, oxidative and nitrosative stress. PLoS One 7: e52850. 23285201 10.1371/journal.pone.0052850 PMC3528649

[21] Liochev SI, Fridovich I (1994) The role of O2.- in the production of HO.: in vitro and in vivo. Free Radic Biol Med 16: 29–33. 8299992 10.1016/0891-5849(94)90239-9

[22] Fradin C, De Groot P, MacCallum D, Schaller M, Klis F, et al (2005) Granulocytes govern the transcriptional response, morphology and proliferation of Candida albicans in human blood. Mol Microbiol 56: 397–415. 15813733 10.1111/j.1365-2958.2005.04557.x

[23] Gillum AM, Tsay EY, Kirsch DR (1984) Isolation of the Candida albicans gene for orotidine-5′-phosphate decarboxylase by complementation of S. cerevisiae ura3 and E. coli pyrF mutations. Mol Gen Genet 198: 179–182. 6394964 10.1007/BF00328721

[24] Lo HJ, Kohler JR, DiDomenico B, Loebenberg D, Cacciapuoti A, et al (1997) Nonfilamentous C. albicans mutants are avirulent. Cell 90: 939–949. 9298905 10.1016/s0092-8674(00)80358-x

[25] Aga E, Katschinski DM, van Zandbergen G, Laufs H, Hansen B, et al (2002) Inhibition of the spontaneous apoptosis of neutrophil granulocytes by the intracellular parasite Leishmania major. J Immunol 169: 898–905. 12097394 10.4049/jimmunol.169.2.898

[26] Meshulam T, Levitz SM, Christin L, Diamond RD (1995) A simplified new assay for assessment of fungal cell damage with the tetrazolium dye, (2,3)-bis-(2-methoxy-4-nitro-5-sulphenyl)-(2H)-tetrazolium-5-carboxanil ide (XTT). J Infect Dis 172: 1153–1156. 7561202 10.1093/infdis/172.4.1153

[27] Haddad SA, Lindegren CC (1953) A method for determining the weight of an individual yeast cell. Appl Microbiol 1: 153–156. 13041190 10.1128/am.1.3.153-156.1953 PMC1056888

[28] Bryan AK, Goranov A, Amon A, Manalis SR (2010) Measurement of mass, density, and volume during the cell cycle of yeast. Proc Natl Acad Sci U S A 107: 999–1004. 20080562 10.1073/pnas.0901851107 PMC2824314

[29] Scudiero DA, Shoemaker RH, Paull KD, Monks A, Tierney S, et al (1988) Evaluation of a soluble tetrazolium/formazan assay for cell growth and drug sensitivity in culture using human and other tumor cell lines. Cancer Res 48: 4827–4833. 3409223

[30] Wellington M, Dolan K, Krysan DJ (2009) Live Candida albicans suppresses production of reactive oxygen species in phagocytes. Infect Immun 77: 405–413. 18981256 10.1128/IAI.00860-08 PMC2612242

[31] Cassatella MA, Gasperini S, Russo MP (1997) Cytokine expression and release by neutrophils. Ann N Y Acad Sci 832: 233–242. 9704051 10.1111/j.1749-6632.1997.tb46251.x

[32] Zavrel M, Majer O, Kuchler K, Rupp S (2011) Transcription factor Efg1 shows a haploinsufficiency phenotype in modulating the cell wall architecture and immunogenicity of Candida albicans. Eukaryot Cell 11: 129–140. 22140230 10.1128/EC.05206-11 PMC3272906

[33] Cheng SC, van de Veerdonk FL, Lenardon M, Stoffels M, Plantinga T, et al. The dectin-1/inflammasome pathway is responsible for the induction of protective T-helper 17 responses that discriminate between yeasts and hyphae of Candida albicans. J Leukoc Biol 90: 357–366. 21531876 10.1189/jlb.1210702 PMC3513931

[34] Behnsen J, Narang P, Hasenberg M, Gunzer F, Bilitewski U, et al (2007) Environmental dimensionality controls the interaction of phagocytes with the pathogenic fungi Aspergillus fumigatus and Candida albicans. PLoS Pathog 3: e13. 17274685 10.1371/journal.ppat.0030013 PMC1790725

[35] Wellington M, Koselny K, Krysan DJ (2012) Candida albicans morphogenesis is not required for macrophage interleukin 1beta production. MBio 4: e00433–00412. 23269828 10.1128/mBio.00433-12 PMC3531805

[36] Kruppa M, Greene RR, Noss I, Lowman DW, Williams DL C. albicans increases cell wall mannoprotein, but not mannan, in response to blood, serum and cultivation at physiological temperature. Glycobiology 21: 1173–1180. 10.1093/glycob/cwr051 PMC3150113 21515585

[37] Donini M, Zenaro E, Tamassia N, Dusi S (2007) NADPH oxidase of human dendritic cells: role in Candida albicans killing and regulation by interferons, dectin-1 and CD206. Eur J Immunol 37: 1194–1203. 17407098 10.1002/eji.200636532

[38] Gantner BN, Simmons RM, Underhill DM (2005) Dectin-1 mediates macrophage recognition of Candida albicans yeast but not filaments. EMBO J 24: 1277–1286. 15729357 10.1038/sj.emboj.7600594 PMC556398

[39] Losse J, Svobodova E, Heyken A, Hube B, Zipfel PF, et al. Role of pH-regulated antigen 1 of Candida albicans in the fungal recognition and antifungal response of human neutrophils. Mol Immunol 48: 2135–2143. 10.1016/j.molimm.2011.07.007 21820180

[40] Soloviev DA, Jawhara S, Fonzi WA (2011) Regulation of innate immune response to Candida albicans infections by alphaMbeta2-Pra1p interaction. Infect Immun 79: 1546–1558. 21245270 10.1128/IAI.00650-10 PMC3067562

