# Supplementary figures and images for: Novel Insight into Neutrophil Immune Responses by Dry Mass Determination of Candida albicans Morphotypes
Source: PLoS One. 2013 Oct 30;8(10):e77993. doi: 10.1371/journal.pone.0077993 (PMC3813559; doi:10.1371/journal.pone.0077993)

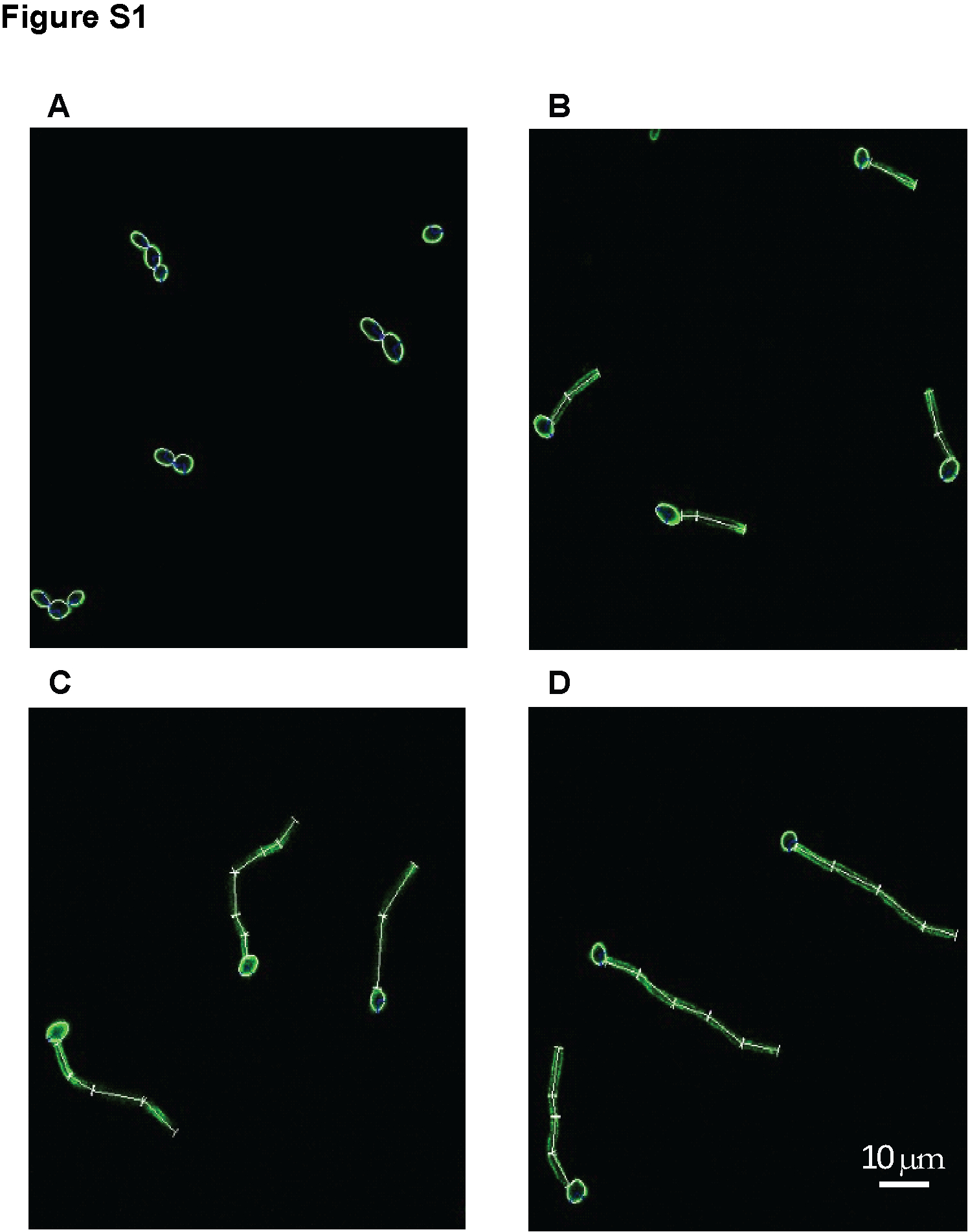

Supplement: Figure S1 — Determination of C. albicans cell surface area. The cell surface area was calculated by measuring the dimensions of immuno-stained C. albicans (anti-Candida antibody). Illustrated here are yeast at 1 h (A) and hyphae at 2 (B), 3 (C) and 4 h (D) after induction, respectively. The overall cell surface area was calculated by using the ellipsoid formula for budding yeasts and the cylinder formula for hyphae. Confocal microscopy (Nikon C1 confocal microscope, NIS-Elements AR, version 3.2.0) was applied and z-stacks were obtained from an average of 50 images to measure the volume of C. albicans cells at different time points. (TIF) [file pone.0077993.s001.tif]

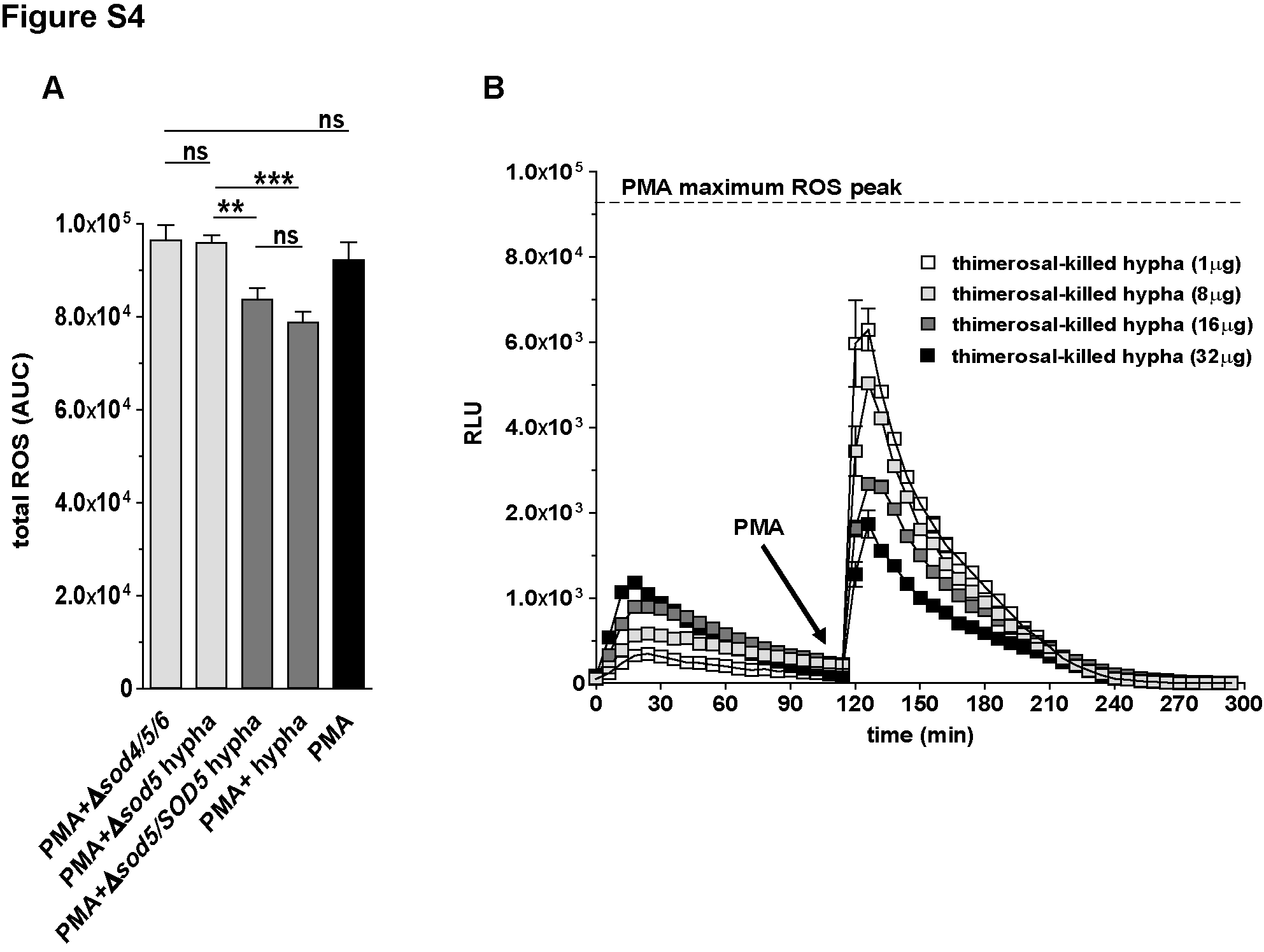

Supplement: Figure S4 — Live and thimerosal-killed C. albicans detoxify neutrophil ROS. Spiking ROS assay: Each column represents AUC corresponding to the total ROS generated by neutrophils over 180 min. Neutrophils (105) were stimulated with PMA (100 nM). After 20 min neutrophils were spiked with 10 µg C. albicans Δsod4/5/6, Δsod5, Δsod5:SOD5, wild-type hypha (A). Relative Light Units (RLUs) correspond to neutrophil ROS in a luminol-based chemiluminescence assay. For each sample 105 neutrophils were infected with different masses of thimerosal-killed C. albicans; after 120 min, the neutrophils were spiked with 100 nM PMA (B). Data are presented as means of three technical replicates ±SD (NS: P>0.05, **P≤0.01 and ***P≤0.001). Masses to MOI conversion for 105 neutrophils: (Hyphae) 1 µg≙0.2, 8 µg≙1.4, 16 µg≙2.7, 32 µg≙5.4. (TIF) [file pone.0077993.s004.tif]
